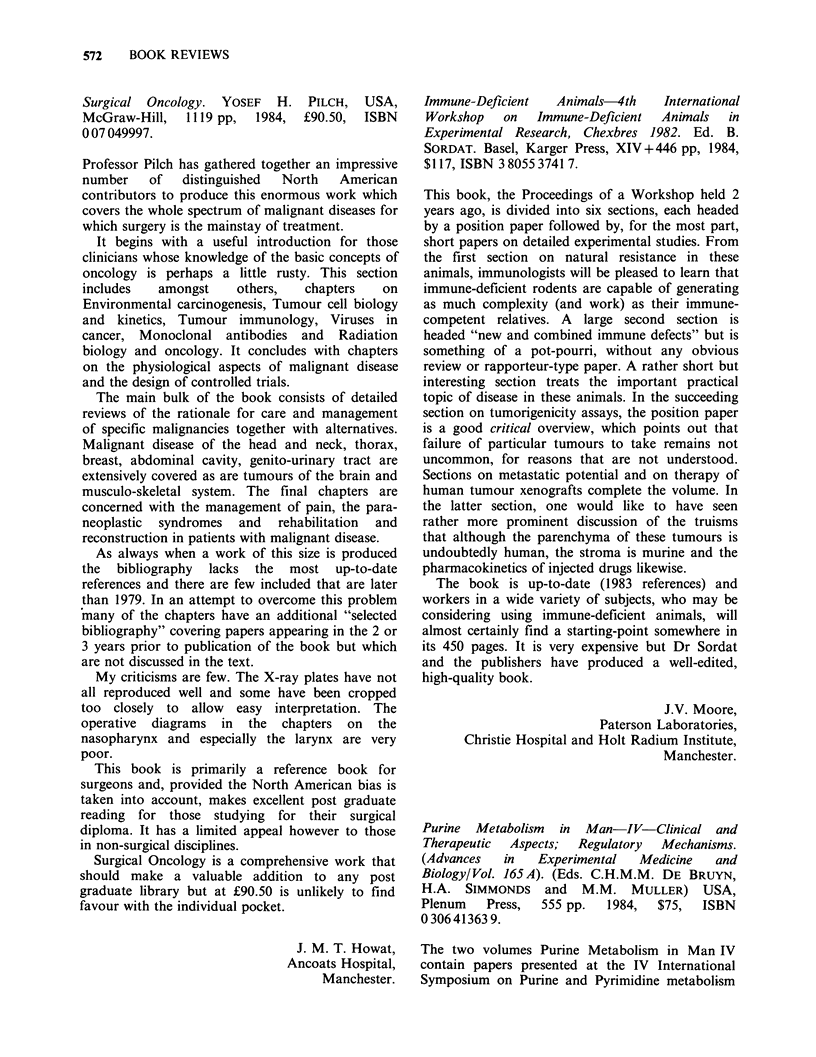# Immune-Deficient Animals—4th International Workshop on Immune-Deficient Animals i Experimental Research, Chexbres 1982

**Published:** 1984-10

**Authors:** J.V. Moore


Manchester.

*Immune-Deficient Animals—4th International Workshop on Immune-Deficient Animals in Experimental Research, Chexbres 1982.* Ed. B. SORDAT. Basel, Karger Press, XIV+446 pp, 1984, $117, ISBN 3 8055 3741 7.

This book, the Proceedings of a Workshop held 2 years ago, is divided into six sections, each headed by a position paper followed by, for the most part, short papers on detailed experimental studies. From the first section on natural resistance in these animals, immunologists will be pleased to learn that immune-deficient rodents are capable of generating as much complexity (and work) as their immune-competent relatives. A large second section is headed "new and combined immune defects" but is something of a pot-pourri, without any obvious review or rapporteur-type paper. A rather short but interesting section treats the important practical topic of disease in these animals. In the succeeding section on tumorigenicity assays, the position paper is a good *critical* overview, which points out that failure of particular tumours to take remains not uncommon, for reasons that are not understood. Sections on metastatic potential and on therapy of human tumour xenografts complete the volume. In the latter section, one would like to have seen rather more prominent discussion of the truisms that although the parenchyma of these tumours is undoubtedly human, the stroma is murine and the pharmacokinetics of injected drugs likewise.

The book is up-to-date (1983 references) and workers in a wide variety of subjects, who may be considering using immune-deficient animals, will almost certainly find a starting-point somewhere in its 450 pages. It is very expensive but Dr Sordat and the publishers have produced a well-edited, high-quality book.

J.V. Moore,
Paterson Laboratories,
Christie Hospital and Holt Radium Institute,
Manchester.